# Active Surveillance for Adverse Events After a Mass Vaccination Campaign With a Group A Meningococcal Conjugate Vaccine (PsA-TT) in Mali

**DOI:** 10.1093/cid/civ497

**Published:** 2015-11-09

**Authors:** Kirsten S. Vannice, Modibo Keita, Samba O. Sow, Anna P. Durbin, Saad B. Omer, Lawrence H. Moulton, Téné M. Yaméogo, Patrick L. F. Zuber, Uma Onwuchekwa, Massambou Sacko, Fabien V. K. Diomandé, Neal A. Halsey

**Affiliations:** 1 Department of International Health, Johns Hopkins Bloomberg School of Public Health, Baltimore, Maryland; 2 Centre pour le Développement des Vaccins, Ministère de la Santé, Bamako, Mali; 3 Emory Vaccine Center and Rollins School of Public Health, Emory University, Atlanta, Georgia; 4 Institut supérieur des sciences de la Santé, Université Polytechnique de Bobo-Dioulasso, Burkina Faso; 5 Department of Essential Medicines and Health Products, World Health Organization, Geneva, Switzerland; 6 World Health Organization, Mali Country Office, Bamako, Mali; 7 Centers for Disease Control and Prevention, Atlanta, Georgia

**Keywords:** vaccine safety, PsA-TT, MenAfriVac, meningitis belt, meningococcal vaccine

## Abstract

***Background.*** The monovalent meningococcal A conjugate vaccine (PsA-TT, MenAfriVac) was developed for use in the “meningitis belt” of sub-Saharan Africa. Mali was 1 of 3 countries selected for early introduction. As this is a new vaccine, postlicensure surveillance is particularly important to identify and characterize possible safety issues.

***Methods.*** The national vaccination campaign was phased from September 2010 to November 2011. We conducted postlicensure safety surveillance for PsA-TT in 40 government clinics from southern Mali serving approximately 400 000 people 1–29 years of age. We conducted analyses with individual-level data and population-level data, and we calculated rates of adverse events using the conditional exact test, a modified vaccine cohort risk interval method, and a modified self-controlled case series method for each outcome of interest, including 18 prespecified adverse events and 18 syndromic categories.

***Results.*** An increased rate of clinic visits for fever within 3 days after vaccination was found using multiple methods for all age groups. Although other signals were found with some methods, complete assessment of all other prespecified outcomes and syndromic categories did not reveal that PsA-TT was consistently associated with any other health problem.

***Conclusions.*** No new safety concerns were identified in this study. These results are consistent with prelicensure data and other studies indicating that PsA-TT is safe. The approach presented could serve as a model for future active postlicensure vaccine safety monitoring associated with large-scale immunization campaigns in low-income countries.

New vaccines, such as those against malaria and dengue, are being developed for use primarily in low-resource countries. Emerging manufacturers provide an increasingly large volume of vaccine products to global programs that will never reach industrialized country markets [1]. Concerted efforts are being made to reduce the time from availability of new vaccines, such as rotavirus and human papillomavirus vaccines, to their introduction in developing countries [2]. The current vaccine pipeline includes products based on complex technologies such as genetic recombination or novel adjuvant systems, and theoretical concerns about the safety of new products indicate the need for postlicensure safety monitoring [3, 4]. Public concern about vaccines can also adversely impact vaccination programs, and having systems in place to monitor safety can help address these concerns with reliable data [5]. Most low-income countries do not have the resources or infrastructure in place for postlicensure safety surveillance.

PsA-TT is a lyophilized conjugate vaccine developed for use in the meningitis belt of sub-Saharan Africa and manufactured by the Serum Institute of India Ltd [6]. Clinical trials in India and West Africa included nearly 12 000 individuals aged 1–29 years, although at the time of licensure <5000 individuals had received the vaccine [7–9]. The World Health Organization (WHO) Global Advisory Committee on Vaccine Safety reviewed clinical trial data and concluded there were no significant safety issues identified from the trials [10], but the committee recommended vaccine safety surveillance postlicensure in countries introducing the vaccine [11, 12].

PsA-TT was initially rolled out in select districts in Burkina Faso, Mali, and Niger in September 2010 with mass vaccination campaigns targeting all individuals 1–29 years of age. The public health benefits of PsA-TT have already been demonstrated by a sharp decline in reported cases of meningococcal disease in the countries where it has been introduced [13–15].

The purpose of this study was to assess the safety of the PsA-TT vaccine in people aged 1–29 years in Mali. Previous studies of PsA-TT utilized passive surveillance systems to assess vaccine safety. Here, we report the results of a novel active vaccine safety surveillance system that we piloted during a rollout campaign in Mali.

## METHODS

### Data Collection

**Figure 1.**
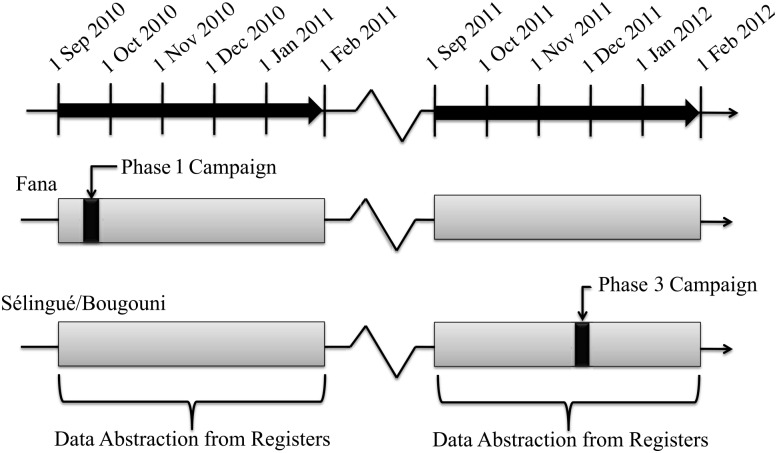
Patient visit dates abstracted before and after the PsA-TT vaccination campaigns (phase 1: 13–20 September 2010; phase 3: 15–24 November 2011). Data were abstracted for visits occurring between 1 September and 31 January of the following year.

A detailed description of how the active surveillance system was designed, risk windows, and how data were collected, abstracted, and processed (unpublished data). In brief, data were abstracted from clinic registers from 40 government health clinics in 3 health districts in southern Mali: Bougouni, Fana, and Sélingué. Data were abstracted for all patient visits occurring in individuals 1–29 years of age between 1 September 2010 and 31 January 2011 and between 1 September 2011 and 31 January 2012 (Figure 1). This data collection period included the first phase of the countrywide vaccination campaign that occurred in Fana (September 2010) and the third phase of the countrywide vaccination campaign that occurred in Bougouni and Sélingué (November 2011). Thirty-seven community clinics (Centres de Santé Communautaire [CSComs]), and 3 referral clinics (Centres de Santé de Référence [CSRefs]) participated. The population of individuals 1–29 years of age under surveillance in our study areas was estimated to be 49 029 in Sélingué, 196 497 in Bougouni, and 154 408 in Fana based on projections from the 2009 census. Data were abstracted from outpatient registers from CSComs and CSRefs and from inpatient registers and obstetric/gynecologic inpatient registers from CSRefs. A data collection team abstracted data directly from the registers into laptops.

In addition to the information collected routinely in the register, we asked the heads of participating health centers during the second data collection period (September 2011–January 2012) to obtain the following information from the patients who attended the clinics during the 10-day November 2011 campaign and for 42 days after the campaign: (1) PsA-TT vaccination status, (2) date of PsA-TT vaccination, and (3) geographic location of PsA-TT vaccination. Visual aids were available to help the patients recollect vaccine status and date of vaccination. No verification of self-reported information was attempted because vaccination cards were not given during this particular campaign.

### Outcomes of Interest

Prespecified health events of interest were identified collectively by study investigators based on biologic plausibility and experience with other vaccines. Each outcome was assigned a prespecified risk window. For individual-based analyses, time since vaccination was used; for population-based analyses, time since the first day of the campaign was used. The prespecified adverse events were abscess at the injection site, anaphylactic shock, cellulitis, convulsions, encephalomyelitis, fever, hypotonia, laryngeal edema, local reactions, meningitis-like syndrome, paralysis, purpura, sepsis, shock, thrombosis, unexplained death, urticaria/itchy rash, and wheezing/bronchospasm. Two control outcomes, trauma and diarrhea, that were not expected to be associated with vaccination were also included.

To limit the number of comparisons for other health events, clinical presentations were collapsed into 18 syndromic categories. Illnesses were assigned to the most appropriate syndromic category when >1 category might apply; for example, measles was classified as infectious rather than dermatologic, and anemia was assigned to the hematologic syndrome.

### Risk Windows

**Figure 2.**
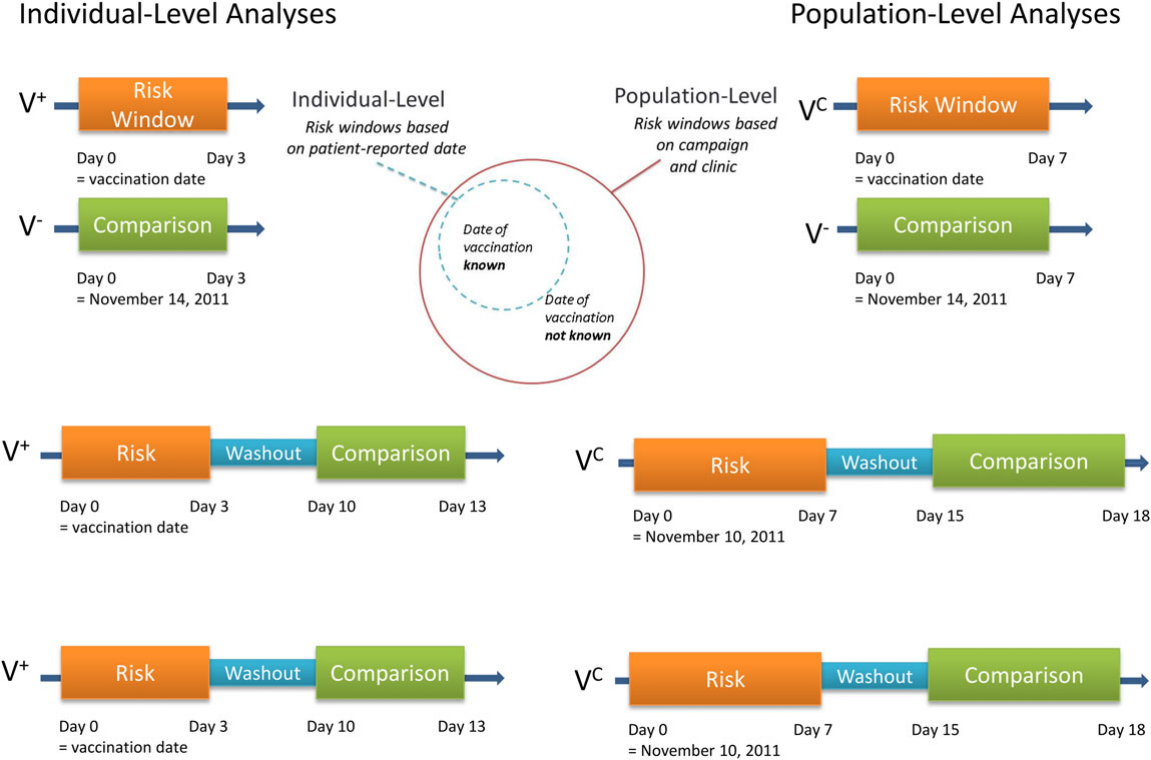
Diagram of methods and populations used in the statistical analyses with risk windows illustrated for fever. “V^+^” denotes vaccine status by self-report, “V^C^” denotes vaccine status by campaign district, and “V^−^” denotes an unvaccinated district. For the individual-level analysis using the conditional exact test (CET), the risk windows were oriented around the vaccination date in the vaccinated population and the median date of vaccine doses administered (18 November 2011) applied to the unvaccinated population. For the population-level analysis using the CET, the risk windows were oriented around the day after the start of the campaign (16 November 2011) for both the vaccinated and unvaccinated population and were extended an additional 3 days. For the individual-level analysis using both the self-controlled case series (SCCS) and self-controlled risk interval (SCRI) analyses, the risk windows were oriented around the date of vaccination with the comparison windows following after a washout period of 7 days. For the population-level analysis using the SCCS and SCRI methods, the risk windows were oriented around the day after the start of the vaccination campaign (16 November 2011) and were extended an additional 3 days, with the comparison windows following after a washout period of 7 days.

Risk windows were developed for biologically plausible time windows following vaccination for both prespecified outcomes and syndromic categories. For individual-level–based analyses (ie, those using individual-level data about vaccination), the risk windows were based on the date of vaccination for those for whom vaccination status/date of vaccination was known, and for population-level analyses (ie, those using population estimates of vaccine coverage to assume vaccination status), the risk windows encompass at least the first 7 days of the vaccine campaign, when most of the doses were given, in addition to the individual-level risk window (Figure 2).

Some risk windows were shorter than originally planned as the date of vaccination was collected for only 6 weeks after the campaign, which did not provide sufficient follow-up data for 42-day risk windows and 42-day control windows (eg, paralysis and purpura risk windows were shortened from 42 days to 21 days). A 7-day washout period was included after the risk window when there was sufficient observation time. Control windows followed risk windows. A major religious holiday 9 days prior to the start of the vaccination campaign precluded using control windows prior to vaccination. Also, individuals who had clinic visits prior to the campaign could not be used for individual analyses as we could not determine if they subsequently received the vaccine during the campaign.

### Statistical Methods

The conditional exact test (CET) was used to estimate the risk of an adverse event in individuals in the vaccinated areas compared with individuals in unvaccinated areas experiencing an event during the predefined risk window (Figure 2). Modified self-controlled case series analyses (SCCS) [16] were used to compare the risk of vaccination occurring among individuals who experienced the specified outcome in defined risk and control windows. Incidence rate ratios (IRRs) estimated from SCCS were estimated using a conditional Poisson regression. To assess the effect of expected seasonal changes on the incidence of fever, the incidence in risk and control windows in the year with the campaign was compared to pseudo-risk and control windows of the same month and days in the off-campaign year and estimated a ratio of IRRs (RIRR) [17]. The RIRR was estimated for Fana and for Sélingué/Bougouni with population-level analyses using the SCCS method. Self-controlled risk interval analysis (SCRI) was used to compare the risk of experiencing an event in defined risk and control windows among individuals who have been vaccinated. IRRs from SCRI were estimated using an unconditional Poisson regression. No correction was performed to account for multiple testing in order to identify possible signals for rare events. Because more than 300 comparisons were conducted, we anticipated some associations due to chance alone. All outcomes were analyzed on an individual level for the patients for whom vaccination status was known and on a population level with vaccination status assumed based on geographic location, because of the high coverage levels. We estimated the attributable risk of fever by subtracting the incidence proportion in the geographically separate unvaccinated population from 18 to 21 November 2011 (the median date of doses administered in the campaign districts), from the incidence proportion among vaccinees in the 3 days following vaccination.

Positive associations by statistical tests between vaccination and syndromic categories were further reviewed for temporal trends among vaccinated individuals and during the entire period of observation to identify possible increased risk in the vaccinated population or around the time of the vaccination campaign, both for the syndromic category and the common illness within the category.

## RESULTS

**Figure 3.**
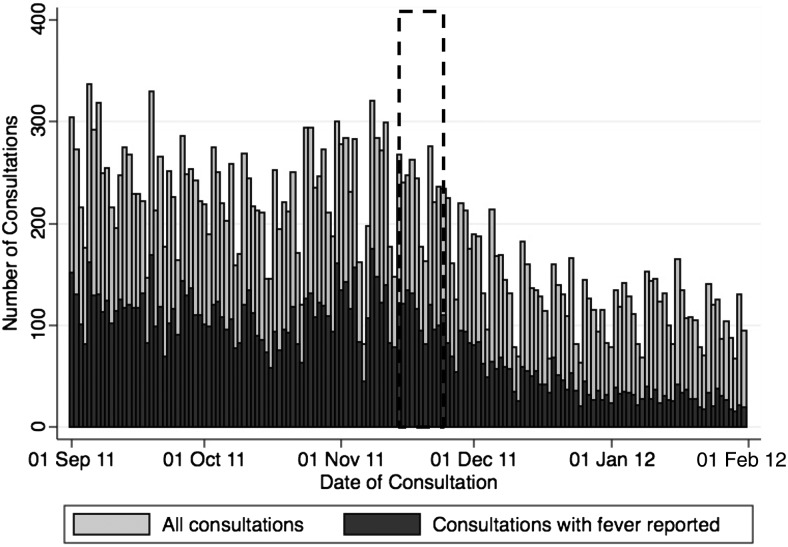
Number of consultations per day for any reason (light bars) and for fever (dark bars) in Sélingué and Bougouni, Mali, 1 September 2011 through 31 January 2012. PsA-TT was administered 15 November–24 November 2011 in the phase 3 campaign (indicated by dotted lines).

No change in seasonal patterns in clinic visits for any cause was associated with the campaigns (Figure 3). A similar seasonal pattern of clinic visits was observed for all clinics during the year the campaign was not conducted in that district (data not shown). The number of consultations for fever per day was highest in September and October, with a subsequent decline in visits, followed by a later peak in early November, prior to the campaign (Figure 3). Date of vaccination was available for 2721 of the 5704 (47.7%) patient visits during and for 42 days after the November 2011 campaign. A total of 4461 (77.7%) patients reported receiving PsA-TT. Five hundred sixty-four (9.8%) reported not receiving PsA-TT; for 718 (12.5%) patients, vaccination status was not captured.

### Prespecified Adverse Events

With the exceptions of fever and convulsions, the majority of prespecified adverse events of interest were reported infrequently (<10 events in the risk/control windows for any analysis). Significantly increased IRRs associated with vaccination (positive associations) were estimated for fever (8 of 9 analyses), convulsions (2 of 9 analyses), and diarrhea (3 of 9 analyses) (Table 1).

**Table 1.** Estimated Incidence Rate Ratios (and 95% Confidence Intervals) for Prespecified Event Analyses

Event	2011 Campaign, Individual Level			2011 Campaign, Population Level			2010 Campaign, Population Level		
	CET	SCCS	SCRI	CET	SCCS	SCRI	CET	SCCS	SCRI
Abscess at injection site	…	…	…	…	1.0 (.1–16.0)	1.0 (.1–16.0)	…	…	…
Anaphylactic shock	…	…	…	…	…	…	…	…	…
Cellulitis	…	…	…	…	…	…	…	…	…
Convulsions	0.7 (.2–2.9)	1.3 (.6–2.9)	0.6 (.2–1.9)	**2.2 (1.1–4.7)**	**1.7 (1.0–2.9)**	0.5 (.2–1.0)	0.8 (.4–1.3)	1.4 (.7–2.8)	1.4 (.7–2.8)
Encephalomyelitis	…	…	…	…	1.0 (.1–16.0)	…	…	…	…
Fever	**1.6 (1.3–1.9)**	**1.4 (1.2–1.6)**	**1.4 (1.2–1.7)**	**1.8 (1.6–2.1)**	**1.6 (1.4–1.8)**	**1.6 (1.4–1.8)**	**0.9 (.8–1.0)**	**1.3 (1.1–1.5)**	**1.3 (1.1–1.5)**
Hypotonia	…	…	…	…	…	…	…	…	…
Laryngeal edema	…	…	…	…	…	…	…	…	…
Local reactions	…	…	…	…	…	…	…	…	…
Meningitis-like	…	…	…	…	0.5 (.0–5.5)	…	1.2 (.2–7.0)	1.5 (.3–9.0)	1.5 (.3–9.0)
Paralysis	…	…	…	…	0.6 (.1–2.8)	1.7 (.2–16.5)	1.9 (.5–7.9)	0.9 (.2–3.0)	1.5 (.4–5.3)
Purpura	…	…	…	…	…	…	…	…	…
Sepsis	…	…	…	…	…	…	…	…	…
Shock	…	…	…	…	…	…	…	…	…
Thrombosis	…	…	…	…	…	…	…	…	…
Unexplained death	…	…	…	…	…	…	…	…	…
Urticaria	3.3 (.4–156.6)	1.4 (.4–4.4)	1.3 (.3–4.7)	0.9 (.4–2.4)	1.0 (.5–2.2)	0.5 (.2–1.3)	1.5 (.7–3.3)	0.6 (.3–1.1)	0.6 (.3–1.1)
Wheezing	0.3 (.0–6.4)	…	…	0.6 (.2–2.4)	…	…	0.8 (.0–15.3)	0.5 (.0–5.5)	0.5 (.0–5.5)
Trauma (control)	0.9 (.6–1.4)	0.9 (.7–1.3)	1.0 (.7–1.5)	1.2 (.9–1.5)	1.0 (.8–1.2)	0.9 (.7–1.2)	**0.6 (.4–.7)**	0.8 (.6–1.0)	0.8 (.6–1.0)
Diarrhea (control)	0.7 (.5–1.1)	1.1 (.8–1.5)	1.0 (.7–1.6)	1.0 (.7–1.4)	1.1 (.8–1.4)	0.9 (.7–1.2)	**1.3 (1.0–1.8)**	**1.4 (1.0–1.9)**	**1.4 (1.0–1.9)**

Bold indicates *P* < .05.

Abbreviations: CET, conditional exact test; SCCS, self-controlled case series; SCRI, self-controlled risk interval.

The IRRs for positive associations for fever ranged from 1.3 (95% confidence interval [CI], 1.1–1.5) to 1.8 (95% CI, 1.6–2.1) (Table 1). The one borderline negative association with fever was obtained using the CET for the population-level analysis during the phase 1 campaign in Fana. When stratified by age group, the positive associations with fever remained across all age groups (Table 2). The significant association with fever remained (IRRs of 1.3 and 1.6) when only a 3-day washout window was used for the self-controlled analyses to reduce the effects of seasonality on clinic visits associated with fever (Table 2). When stratified by sex, the IRR was 2.1 (95% CI, 1.7–2.6) among males living in a vaccinated district compared with males living in an unvaccinated district, and 2.0 (95% CI, 1.6–2.5) among females living in a vaccinated district compared with females living in an unvaccinated district. There was also a significant association among males in the individual-level analyses (IRR, 2.0 [95% CI, 1.5–2.7]). Among individuals who received the vaccine and for whom a vaccination date was available, there were more episodes of fever in the risk window compared with the control window. Fever episodes decreased over time since the campaign start when looking at all patient consultations, regardless of vaccination status (Figure 2). Fewer episodes of fever were recorded in the comparison district where vaccine was not given (Fana) during the same time period. The RIRR for fever consultations during the vaccinated dates compared with the same dates in the comparison periods using population-level SCCS analyses was 1.64 (95% CI, 1.39–1.94) for Sélingué and Bougouni, and 1.35 (95% CI, 1.14–1.61) for Fana. The RIRR remained significant when stratified by age group (Supplementary Material). The attributable risk of fever was 6 cases of clinically relevant fever per 10 000 vaccinees.

**Table 2.** Number of Consultations for Fever and Convulsions in Individuals Receiving PsA-TT During the 2011 Campaign (Individual-Level Analysis), Stratified by Age Group

Outcome and Age Group	CET			SCCS			SCRI		
	No. of Events MAV (+) Individuals	No. of Events MAV (−) Individuals	Individual-Level IRR (95% CI)	No. of Events MAV Risk Window	No. of Events MAV Control Window	Individual-Level IRR (95% CI)	No. of Events MAV Risk Window	No. of Events MAV Control Window	Individual-Level IRR (95% CI)
Convulsions									
1–4 y	5	4	0.8 (.2–4.2)	13	15	0.9 (.4–1.8)	5	10	0.5 (.2–1.5)
5–14 y	0	0	…	1	2	0.5 (.0–5.5)	0	1	…
15–29 y	0	0	…	0	3	…	0	3	…
Fever									
1–4 y	157	64	**1.6 (1.2–2.2)**	225	162	**1.4 (1.1–1.7)**	123	157	**1.3 (1.0–1.6)**
5–14 y	122	35	**2.3 (1.6–3.5)**	141	100	**1.4 (1.1–1.8)**	121	85	**1.4 (1.1–1.9)**
15–29 y	67	36	1.2 (.8–1.9)	100	62	**1.6 (1.2–2.2)**	69	49	**1.4 (1.0–2.1)**

Self-controlled methods used a modified washout of 3 days in this analysis. Bold indicates *P* < .05.

Abbreviations: CET, conditional exact test; CI, confidence interval; IRR, incidence rate ratio; MAV, group A meningococcal vaccine (PsA-TT); SCCS, self-controlled case series; SCRI, self-controlled risk interval.

Eighty percent of all convulsions occurred among those aged 1–4 years. A positive association with convulsions was found in 2 of the initial 9 analyses (Table 1). There was no significant association with convulsions using individual-level analyses when stratified by age using any of the methods including modification of washout window length (Table 2). There was a consistent decrease in the number of consultations for convulsions regardless of vaccination status during and after the vaccination campaign (data not shown).

The incidence of diarrhea was highest in those aged 1–4 years and decreased during the month of October (data not shown) in Fana. The higher incidence rates of diarrhea in the risk vs control windows resulted in positive associations during the 2010 phase 1 campaign in Fana, ranging from IRR of 1.3 (95% CI, 1.0–1.8) to 1.4 (95% CI, 1.0–1.9) (Table 1). Aside from those described above, no other positive associations were noted for either the 2010 phase 1 campaign or 2011 phase 3 campaign.

A negative association was found for 1 of 9 analyses for trauma (Table 1). The IRR of 0.6 (95% CI, .4–.7) was also obtained using the CET for the population-level analysis during the phase 1 campaign. No significant IRRs were found for abscess at the site of injection, encephalomyelitis, meningitis-like syndrome, paralysis, urticaria, and wheezing.

### Syndromic Categories

Significant positive associations (increased risk) or negative associations (decreased risk) were estimated in >3 of the 9 analyses for the categories of ear/nose/throat, gastrointestinal, hematologic, infectious, neurologic, other, and respiratory (Supplementary Material). Upon further investigation, there was no evidence of consistency across analyses or increased risk in the 1–2 days following vaccination for individuals with known vaccination status or temporally associated with the vaccination campaign in the entire population.

## DISCUSSION

No unexpected safety concerns were identified in this study. An elevated risk for fever in the first 3 days and 7 days after vaccination was detected, although the estimated attributable risk was small. Fever is a recognized complication of other polysaccharide protein conjugate vaccines [18, 19], and the association with PsA-TT is biologically plausible. We did not observe a consistent pattern of increased rate of convulsions in most analyses, but we cannot rule out the possibility that the vaccine predisposed recipients in the 1- to 4-year age group to a small increase in the rate of febrile seizures, as has been seen with pneumococcal conjugate vaccines coadministered with other vaccines [20]. Aside from fever, we believe the other estimated significant associations may be explained by 1 or more of the following: (1) chance occurrences associated with the multiple tests performed, (2) declining disease incidence in the control periods due to seasonal patterns of illness, or (3) higher disease incidence in some districts compared with geographically separated districts due to better access to health facilities or underlying differences in disease incidence. The long risk windows assigned to many of the syndromic categories made them particularly susceptible to seasonal bias.

Our results are consistent with PsA-TT safety studies conducted in Burkina Faso and Niger using different surveillance strategies in which enhanced passive surveillance did not detect any safety concerns for the prespecified adverse events [21, 22]. The adverse events reported most frequently by clinicians were fever, headache, gastrointestinal disorders, and local reactions. Our study did not identify many local reactions, most likely due to the fact that most local reactions were not sufficiently severe to warrant attendance at a clinic. In the enhanced surveillance studies, all reports were for events that occurred within 16 days of vaccination and highlight recognized patterns of reporting events closer to the exposure date [21]. The authors noted that reporting rates were much lower than background rates with the exception of local reactions. Passive surveillance in Niger found fever, gastrointestinal disorders, convulsions, headache, and urticaria to be reported most frequently, although the authors note the likelihood that many reported events (particularly of fever and convulsions) were coincidental infectious diseases and likely caused by malaria, given that the campaign occurred in September, during the malaria season [22]. During clinical trials of PsA-TT, proportions of patients with fever following a primary dose of the vaccine ranged from 0% (N = 24 males aged 18–35 years in India) [7], 3.0% within 4 days (N = 604 aged 2–29 years in Mali) [8], 4.0% within 4 days (N = 201 aged 12–23 months in Mali) [8], and 6.5% within 4 days (N = 169 aged 2–10 years in India) [9].

When comparing the incidence of illnesses between vaccinated and control populations, we often saw reverses in the direction of associations depending on which district received vaccine, suggesting that the differences in health-seeking behaviors or disease patterns between the sites were responsible for the associations, rather than a protective or enhanced effect of the vaccine. This highlights the problems with using comparison groups for controls that are geographically or otherwise different from the vaccinated populations due to differences in health, health-seeking behaviors, or healthcare delivery.

There were several limitations to our study. We did not capture illnesses in individuals who experienced adverse health outcomes but did not attend a clinic or died before reaching one. The register diagnoses were not validated, but we would not anticipate differences in the reliability of these diagnoses during the observational periods. We treated each incident visit as a different individual, although patients may have visited multiple times during the data collection period. Because we could not follow individuals over time, we assumed that individuals who experienced an event in one window were at risk during the entire observation period. The recalled date of vaccination may also be incorrect, which could have affected associations with illnesses with short risk windows.

## CONCLUSIONS

This study reports data from an active surveillance system on the largest population receiving PsA-TT to date and serves as a pilot of a novel method for active vaccine safety surveillance in low-income countries. We believe the self-controlled methods have utility in this setting, particularly for immunizations given in the routine schedule that are not clustered at one point in time. Caution should be exercised when looking at outcomes with a strong seasonal pattern, even over short risk and control windows. Our study also allowed for the possibility of identifying unanticipated adverse events through analysis of syndromic categories and to test associations that may have arisen from passive surveillance or public concern, but were not included in the prespecified list. We used multiple methods and populations to allow us to look for consistencies across population, place, and time. Active surveillance studies conducted in rural settings using routinely collected data would be greatly improved by overall health system strengthening, including better diagnostic capacity, nominal vaccination registers (or other methods by which to verify individual vaccination status), and electronically collected data for ease of processing and analysis. In the absence of these lofty goals, sufficient resources and lead time for planning and training are critical.

## Supplementary Data

Supplementary materials are available at *Clinical Infectious Diseases* online (http://cid.oxfordjournals.org). Supplementary materials consist of data provided by the author that are published to benefit the reader. The posted materials are not copyedited. The contents of all supplementary data are the sole responsibility of the authors. Questions or messages regarding errors should be addressed to the author.

Supplementary Data
