# Supplementary material for: Active Surveillance for Adverse Events After a Mass Vaccination Campaign With a Group A Meningococcal Conjugate Vaccine (PsA-TT) in Mali
Source: Clin Infect Dis. 2015 Nov 9;61(Suppl 5):S493–500. doi: 10.1093/cid/civ497 (PMC4639483; doi:10.1093/cid/civ497)
Supplement: Supplementary Data [file supp_61_suppl-5_S493__index.html]

Supplementary Data 

# Active Surveillance for Adverse Events After a Mass Vaccination Campaign With a Group A Meningococcal Conjugate Vaccine (PsA-TT) in Mali

## Supplementary Data

Supplementary Data

- Supplementary File 1 - docx file
- Supplementary File 2 - docx file
