# Supplementary material for: Active Surveillance for Adverse Events After a Mass Vaccination Campaign With a Group A Meningococcal Conjugate Vaccine (PsA-TT) in Mali
Source: Clin Infect Dis. 2015 Nov 9;61(Suppl 5):S493–500. doi: 10.1093/cid/civ497 (PMC4639483; doi:10.1093/cid/civ497)
Supplement: Supplementary Data [file supp_civ497_civ497supp_file1.docx]

**Table S2-1.** Individual-level and population-level risk windows for pre-specified adverse health outcomes of interest.

| **Pre-Specified Health Outcome** | **Individual-level Risk Window (time since vaccination)** | **Population-level Risk Window**  **(time since start of vaccination campaign)** |
| --- | --- | --- |
| Abscess at injection site | 0 – 7 days | 0 – 14 days |
| Anaphylaxis | 0 – 72 hours | 0 – 10 days |
| Cellulitis | 1 – 7 days | 1 – 21 days† |
| Encephalomyelitis | 1– 28 days*† | 1 – 35 days† |
| Fever | 0 – 72 hours | 1 – 7 days |
| Hypotonia | 0 – 48 hours | 1 – 9 days |
| Laryngeal edema | 0 – 72 hours | 0 – 10 days |
| Local reactions | 0 – 7 days | 0 – 12 days |
| Meningitis-like syndrome | 1 – 28 days*† | 1 – 35 days† |
| Paralysis | 1 – 42 days*† | 1 – 49 days† |
| Purpura | 0 – 28 days*† | 0 – 35 days |
| Seizures | 0 – 72 hours | 0 – 7 days |
| Sepsis | 0 – 7 days | 0 – 14 days |
| Shock | 0 – 7 days | 0 – 14 days |
| Thrombosis | 1 – 28 days*† | 1 – 35 days† |
| Urticaria | 0 – 72 hours | 0 – 10 days |
| Unexplained death | 0 – 72 hours | 0 – 10 days |
| Wheezing | 0 – 72 hours | 0 – 10 days |
| Other serious neurological disorders | 1 – 42 days*† | 1 – 49 days† |
| Trauma (Control outcome) | 0-7 days  0-28 days* | 0-14 days  0-35 days† |
| Diarrhea (Control outcome) | 0-7 days  0-28 days* | 0-14 days  0-35 days† |

*1-21 day risk window used for self-controlled methods due to limitations in vaccination date. †No wash out period was used between risk and control windows.

**Figure S2-1.** Ratios of population-level SCCS IRRs for fever for Sélingué/Bougouni (SB) and Fana (F) for all ages (blue), 1-4 years (green), 5-14 years (orange), and 15-29 years (red). Lighter colors are S/B, darker colors are F.
