# Supplementary material for: Active Surveillance for Adverse Events After a Mass Vaccination Campaign With a Group A Meningococcal Conjugate Vaccine (PsA-TT) in Mali
Source: Clin Infect Dis. 2015 Nov 9;61(Suppl 5):S493–500. doi: 10.1093/cid/civ497 (PMC4639483; doi:10.1093/cid/civ497)
Supplement: Supplementary Data [file supp_civ497_civ497supp_file2.docx]

**Table S3-1**. Individual-level and population-level risk windows for syndromic categories.

| **Syndrome Category** | **Individual-level**  **Risk Window** | **Population-level**  **Risk Window** |
| --- | --- | --- |
| Allergic | 0 – 72 hours | 0 – 10 days |
| Cardiovascular | 0 – 42 days*† | 1 – 49 days† |
| Dermatological | 0 – 72 hours | 0 – 10 days |
| Endocrine | 0 – 42 days*† | 1 – 49 days† |
| ENT | 0 – 42 days*† | 1 – 49 days† |
| Gastrointestinal | 0 – 7 days | 0 – 14 days |
| Genital-Urinary | 0 – 42 days*† | 1 – 49 days† |
| Hematologic | 0 – 42 days*† | 1 – 49 days† |
| Infectious | 0 – 14 days | 0 – 21 days |
| Musculoskeletal | 0 – 42 days*† | 1 – 49 days† |
| Neurological | 0 – 42 days*† | 1 – 49 days† |
| Non-Specific | 0 – 7 days | 0 – 14 days |
| Obstetric | 0-14 days | 0 – 21 days |
| Odonto | 0 – 42 days*† | 1 – 49 days† |
| Other | 0 – 42 days*† | 1 – 49 days† |
| Renal | 0 – 14 days | 0 – 21 days |
| Respiratory | 0 – 14 days | 0 – 21 days |
| Trauma | 0 – 3 days | 0 – 10 days |

*1-21 day risk window used for self-controlled methods due to limitations in vaccination date.

†No wash out window

**Table** **S3-2.** Estimated incidence rate ratios for syndromic categories using the conditional exact test, self-controlled case series, self-controlled risk interval analyses for individual-level (2011 Campaign) and population-level (2010 and 2011 Campaigns) analyses. Bold indicates

|  | **2011 Campaign**  **Individual Level** | | | **2011 Campaign**  **Population Level** | | | **2010 Campaign**  **Population Level** | | |
| --- | --- | --- | --- | --- | --- | --- | --- | --- | --- |
| **Syndromic Category** | **CET** | **SCCS** | **SCRI** | **CET** | **SCCS** | **SCRI** | **CET** | **SCCS** | **SCRI** |
| Allergic | 2.6  (0.3-130.4) | 4.0  (0.4-35.8) | 4.0  (0.4-35.8) | 3.1  (0.4-148.7) | 1.7  (0.4-7.0) | 1.7  (0.4-7.0) | 1.9  (0.5-7.9) | 0.6  (0.2-1.7) | 1.5  (1.0-2.1) |
| Cardiovascular | 0.9  (0.3-2.4) | 0.7  (0.3-1.5) | 0.5  (0.1-1.6) | 1.4  (0.7-3.4) | 0.8  (0.4-1.6) | - | 0.7  (0.4-1.2) | 1.4  (0.7-2.7) | **1.6**  **(1.2-2.1)** |
| Dermatologic | 0.8  (0.4-1.6) | 1.4  (0.8-2.4) | 1.5  (0.8-3.1) | 0.9  (0.6-1.4) | 1.1  (0.7-1.5) | 1.1  (0.7-1.5) | 0.8  (0.6-1.2) | 0.7  (0.5-1.0) | 1.1  (1.0-1.2) |
| Endocrine | 0.3  (0.0-6.4) | - | - | 0.6  (0.0-8.7) | 0.3  (0.1-1.6) | - | 1.2  (0.2-7.0) | - | 1.4  (0.8-2.6) |
| Ear/Nose/  Throat | 1.2  (1.0-1.6) | **1.7**  **(1.3-2.2)** | **1.8**  **(1.3-2.4)** | **1.6**  **(1.3-2.0)** | **1.3**  **(1.0-1.6)** | **1.3**  **(1.0-1.6)** | **0.7**  **(0.6-0.9)** | **1.6**  **(1.2-2.0)** | 0.7  (0.1-4.0) |
| Gastro-intestinal | 1.1  (0.9-1.2) | **1.5**  **(1.3-1.7)** | **1.6**  **(1.3-1.8)** | **1.4**  **(1.2-1.5)** | **1.7**  **(1.6-1.9)** | **1.4**  **(1.3-1.6)** | **0.8**  **(0.7-0.9)** | 1.1 (1.0-1.3) | **1.3**  **(1.1-1.6)** |
| Genital-Urinary | **0.7**  **(0.5-0.9)** | 1.3  (0.9-1.7) | 1.4  (0.9-2.0) | 1.0  (0.8-1.2) | **0.7**  **(0.6-0.9)** | **0.7**  **(0.6-0.9)** | 1.1  (0.9-1.4) | 1.2  (0.9-1.5) | - |
| Hematologic | 1.2  (0.9-1.8) | **2.6**  **(1.9-3.6)** | **3.0**  **(1.9-4.7**) | **2.1**  **(1.6-2.8)** | **2.0**  **(1.5-2.7)** | **2.0**  **(1.5-2.7)** | **0.5**  **(0.4-0.6)** | **1.3**  **(1.0-1.8)** | 0.9  (0.9-1.1) |
| Infectious | 1.1  (1.0-1.1) | **1.8**  **(1.7-2.0)** | **1.9**  **(1.7-2.1)** | **1.3**  **(1.3-1.4)** | **2.1**  **(2.0-2.3)** | **1.8**  **(1.6-2.0)** | **0.8**  **(0.8-0.9)** | **0.8**  **(0.7-0.8)** | 0.8  (0.6-1.0) |

**Table S3-2 Continued**

|  | **Phase 3 – Individual Level** | | | **Phase 3 – Population Level** | | | **Phase 1 – Population Level** | | |
| --- | --- | --- | --- | --- | --- | --- | --- | --- | --- |
| **Syndromic Category** | **CET** | **SCCS** | **SCRI** | **CET** | **SCCS** | **SCRI** | **CET** | **SCCS** | **SCRI** |
| Musculo-skeletal | 0.7  (0.5-1.1) | **2.1**  **(1.3-3.3)** | **3.8**  **(1.8-7.9)** | 1.2  (0.8-1.8) | 1.0  (0.7-1.5) | 1.0  (0.7-1.5) | 1.1  (0.8-1.5) | 1.5  (1.0-2.1) | 1.5  (1.0-2.1) |
| Neurologic | 0.8  (0.6-1.2) | **2.2**  **(1.5-3.1)** | **1.8**  **(1.2-2.9)** | **1.4**  **(1.0-1.8)** | **1.8**  **(1.3-2.5)** | **1.8**  **(1.3-2.5)** | 0.9  (0.7-1.1) | **1.6**  **(1.2-2.1)** | **1.6**  **(1.2-2.1)** |
| Non-Specific | **1.3**  **(1.1-1.4)** | **1.7**  **(1.6-1.9)** | **1.7**  **(1.6-2.0)** | **1.5**  **(1.4-1.7)** | **1.9**  **(1.8-2.0)** | **1.9**  **(1.8-2.0)** | **0.8**  **(0.8-0.9)** | 1.1  (1.0-1.2) | 1.1  (1.0-1.2) |
| Obstetric | 0.5  (0.2-1.1) | 1.3  (0.7-2.5) | 1.0  (0.5-2.1) | 1.0  (0.6-1.8) | **1.7**  **(1.0-2.8)** | **1.7**  **(1.0-2.8)** | 1.3  (0.7-2.2) | 1.4  (0.8-2.6) | 1.4  (0.8-2.6) |
| Odontological | 1.0  (0.1-11.9) | - | - | 1.3  (0.2-13.9) | 2.3  (0.3-20.5) | 2.3  (0.3-20.4) | 0.8  (0.1-5.5) | 0.7  (0.1-4.0) | 0.7  (0.1-4.0) |
| Other | **0.8**  **(0.6-1.0)** | 1.2  (0.9-1.5) | 1.2  (0.9-1.6) | **1.2**  **(1.0-1.5)** | **1.3**  **(1.1-1.6)** | **1.3**  **(1.1-1.6)** | 1.0  (0.8-1.2) | **1.3**  **(1.1-1.6)** | **1.3**  **(1.1-1.6)** |
| Renal | 0.3  (0.0-6.4) | - | - | 0.1  (0.0-1.1) | 0.1  (0.0-1.2) | 0.2  (0.0-1.7) | 3.2  (0.2-187.6) | - | - |
| Respiratory | 1.0  (0.9-1.2) | **1.4**  **(1.3-1.7)** | **1.6**  **(1.4-2.0)** | **1.3**  **(1.1-1.5)** | **1.5**  **(1.3-1.6)** | **1.5**  **(1.3-1.6)** | **0.8**  **(0.7-0.9)** | 0.9  (0.8-1.1) | 0.9  (0.9-1.1) |
| Trauma | 0.8  (0.5-1.4) | 0.9  (0.6-1.3) | 1.1  (0.7-1.7) | 1.1  (0.9-1.6) | 0.9  (0.7-1.2) | 1.0  (0.8-1.2) | **0.5**  **(0.4-0.7)** | 1.0  (0.7-1.5) | 0.8  (0.6-1.0) |

**Table S3-3.** Summary of in-depth investigation of select pre-specified events and syndromic categories and interpretations and conclusions**.**

| **Event/Outcome** | **Consistency across analyses** | **Visible peak around vaccination or campaign** | **Seasonal trend** | **Conclusion** |
| --- | --- | --- | --- | --- |
| Fever | Strong consistency | No | Strong seasonal trend | Likely association due to the significant effect estimated with shorter washout period. |
| Convulsions | Weak consistency | No | Strong seasonal trend | Unlikely association and hard to disassociate from malaria-related events |
| Ear/Nose/Throat | Strong consistency | No | Strong seasonal trend | Unlikely association |
| Gastrointestinal | Strong consistency | No | Strong seasonal trend | Unlikely association |
| Hematologic | Strong consistency | No | Moderate seasonal trend | Unlikely association and hard to disassociate from malaria-related events |
| Infectious | Moderate consistency | No | Strong seasonal trend | Unlikely association and hard to disassociate from malaria-related events |
| Neurologic | Strong consistency | No | Minor/moderate seasonal trend | Unlikely association and hard to disassociate from malaria-related events |
| Non-Specific | Strong consistency | No | Strong seasonal trend | Unlikely association and hard to disassociate from malaria-related events |
| Other | Strong consistency | No | Minor/no seasonal trend | Unlikely association |
| Respiratory | Moderate consistency | No | Strong seasonal trend | Unlikely association |

*Summary Text*

*Ear/Nose/Throat Category*

In six of nine analyses, the ear/nose/throat (ENT) category was significantly positively associated with vaccination; in one of nine, it was significantly negatively associated with vaccination (conditional exact test, Phase I campaign). The magnitude of incidence rate ratios ranged between 0.7 (95%CI 0.6-0.8) and 1.8 (95%CI 1.3-2.4) (Table 3-5). When looking at the frequency of ENT diagnoses among those vaccinated by day since vaccination, the decreasing trend continued consistently for 42 days. When examined in the vaccinated areas regardless of vaccination status, there was no unexpected peak in cases around the time of the campaign or in the days that follow that were inconsistent with the general trend from September to February. Of all of the ENT diagnoses during the Phase III campaign in the vaccinated areas, 86% were vertigo. The frequency of vertigo cases among those vaccinated decreases over at least 21 days following vaccination. An examination of the trend of vertigo cases among patients regardless of vaccination status did not suggest an unexpected trend or association with the vaccination campaign.

*Gastrointestinal Category*

In six of nine analyses, the gastrointestinal category was significantly positively associated with vaccination; in one of nine, it was significantly negatively associated with vaccination (conditional exact test, Phase I campaign). The magnitude of incidence rate ratios ranged between 0.8 (95%CI 0.7-0.9) and 1.7 (95%CI 1.6-1.9) (Table 3-5). When looking at the frequency of gastrointestinal diagnoses among those vaccinated by day since vaccination, the decreasing trend continued consistently for 42 days. When examined in the vaccinated areas regardless of vaccination status, there is a slight increase in cases starting in late October that declines during the months of November and December that plateaus in mid-December through the end of the observation period. The most common (63%) of gastrointestinal diagnoses was vomiting. Further examination of vomiting suggested a small peak of vomiting episodes from a few days after receiving vaccine to about one week after vaccination, followed by a steady decline among vaccinees. When vomiting cases were examined among all patients regardless of vaccination status over a five-month period, vomiting cases increased during the end of October 2011 to a peak frequency on November 8, followed by a steady decline through the end of December. One other isolated spike of cases occurred on November 20, in the middle of the vaccination campaign.

*Hematologic Category*

In six of nine analyses, the Hematologic category was significantly positively associated with vaccination; in one of nine, it was significantly negatively associated with vaccination (conditional exact test, Phase I campaign). The magnitude of incidence rate ratios ranged between 0.5 (95%CI 0.4-0.6) and 3.0 (95%CI 1.9-4.7) (Table 3-5). There was a declining trend in hematologic diagnoses among vaccinees over the 42 days following vaccination. There was no apparent peak or inconsistency in the frequency of hematologic cases around the dates of the vaccine campaign, and the frequency declined from a peak in early October to lower levels in mid-December. Seventy-five percent of all hematologic cases were anemia. An analysis of anemia diagnoses among vaccinees shows a general decline since vaccination, which mimicked a general decline in all patients between the beginning of October and the end of the observation period.

*Infectious*

In five of the nine analyses, the infectious category was significantly positively associated with vaccination; in two of nine, it was significantly negatively associated with vaccination (conditional exact test and self-controlled case series, Phase I campaign). The magnitude of incidence rate ratios ranged from 0.8 (95%CI 0.8-0.9) and 2.1 (95%CI 2.0-2.3) (Table 3-5). There was a consistent declining trend of infectious diagnoses in the 42 days since vaccination. Between September 2011 and November 2012, there are many infectious diagnoses. The high frequency began to decline around November 8 and the frequency decreased over the following months through the end of the observation period. The infection syndromic category consisted predominantly of malaria (90%) and the same trends were seen when confined to malaria diagnoses.

*Neurologic*

In seven of the nine analyses, the Neurologic category was significantly positively associated with vaccination. The magnitude of incidence rate ratios ranged from 1.4 (95%CI 1.0-1.8) and 2.2 (95%CI 1.5-3.1) (Table 3-5). The frequency of neurologic cases was fairly consistent over the 42 days following vaccination, with a possible peak between 7 and 13 days after vaccination. There were two small peaks in early October and mid-November, around the start of the vaccination campaign. Of all neurologic cases, 78% were cases of convulsions. A review of the temporal progression of convulsions found no increase in cases in the 72 hours after vaccination (our risk window for this pre-specified event) and no gross aberration in the general trend of convulsion cases from September 2011 to February 2012.

*Respiratory*

In five of the nine analyses, the Respiratory category was significantly positively associated with vaccination; in one of nine, it was significantly negatively associated with vaccination (conditional exact test, Phase I campaign). The magnitude of incidence rate ratios ranged from 0.8 (95%CI 0.7-0.9) and 1.6 (95%CI 1.4-2.0) (Table 3-5). Vaccinees with a respiratory diagnosis declined gradually over time since vaccination. This was also seen in the larger trend of decreasing respiratory diagnoses from September 2011 to February 2012 in patients regardless of vaccination status. Cough was the most common respiratory diagnosis (64%), followed by dyspnea (56%). Cough diagnoses followed a steady decline over time following vaccination, also reflected in the decreased frequency of cough during the 2011-2012 observation period.

*Non-Specific*

In six of nine analyses, the Non-Specific category was significantly positively associated with vaccination; in one of nine, it was significantly negatively associated with vaccination (conditional exact test, Phase I campaign). The magnitude of incidence rate ratios ranged between 0.8 (95%CI 0.8-0.9) and 1.9 (95%CI 1.8-2.0) (Table 3-5). The frequency of consultations for non-specific diagnoses decreased consistently over the 42 days since vaccination. Similar to the analysis done for infectious diagnoses, there was a strong decline in patient visits for non-specific symptoms between the first week of November and the beginning of January. Seventy-five percent of all non-specific diagnoses were fever, which declined consistently over the 42 days since vaccination among vaccinated individuals, and all cases of declined from the end of October to the beginning of January regardless of vaccination status.

*Other*

In five of the nine analyses, the Other category was significantly positively associated with vaccination; in one of nine, it was significantly negatively associated with vaccination (conditional exact test, Phase III campaign). The magnitude of incidence rate ratios ranged from 0.8 (95%CI 0.6-1.0) and 1.3 (95%CI 1.1-1.6) (Table 3-5). The frequency of cases in the Other category did not vary drastically during the 42 days since vaccination among vaccinated individuals or over the course of the 2011-2012 observation period. The most frequent diagnosis included in the Other syndromic category was malnutrition (37%), followed by animal/insect bites (13%). Malnutrition diagnoses showed no peak around the time of vaccination among vaccinees or around the vaccination campaign among all patients.

**Table S3-4.** Number (percent) of outcomes for syndromic categories investigated in-depth constituting 5% or greater of the total from the beginning of Phase III campaign to 42 days after the end of the campaign in the vaccinated districts.

| **Syndromic Category** | **Outcome (>5%) in Syndromic Category** | **Number (%)** |
| --- | --- | --- |
| ENT | All ENT | 312 (100) |
|  | Vertigo | 269 (86) |
| Gastrointestinal | All Gastrointestinal Events | 2497 (100) |
|  | Vomiting | 1581 (63) |
|  | Abdominal Pain | 693 (28) |
|  | Diarrhea | 470 (19) |
| Hematologic | All Hematologic Events | 219 (100) |
|  | Anemia | 165 (75) |
|  | Splenomegaly | 25 (11) |
|  | Contusion | 10 (5) |
| Infectious | All Infectious Events | 5102 (100) |
|  | Malaria (all) | 4588 (90) |
|  | Severe malaria | 1909 (37) |
|  | Typhoid Fever | 338 (7) |
| Neurologic | All Neurologic Events | 160 (100) |
|  | Convulsions | 125 (78) |
| Non-Specific | All Non-Specific Events | 4286 (100) |
|  | Fever | 3207 (75) |
|  | Headache | 1159 (27) |
|  | Loss of Appetite | 320 (7) |
|  | Stiffness/Ache | 229 (5) |
|  | Chills | 221 (5) |
| Other | All Other Events | 336 (100) |
|  | Malnutrition | 124 (37) |
|  | Animal/insect | 45 (13) |
|  | Nothing Evident/No illness | 32 (10) |
|  | Dental-related | 29 (9) |
| Respiratory | All Respiratory Events | 1279 (100) |
|  | Cough | 819 (64) |
|  | Dyspnea | 713 (56) |
|  | ARI | 442 (35) |
|  | Cold | 167 (13) |
|  | Pneumonia | 148 (12) |
|  | Angina | 72 (6) |

**Figure S3-1a.**

Number of Ear/Nose/Throat and vertigo consultations by days since vaccination among those vaccinated and with vaccination date available (Phase III Campaign).

**Figure S3-1b.**

Number of Ear/Nose/Throat and vertigo consultations by calendar date among those living in vaccinated districts regardless of vaccination status (Phase III Campaign).

**Figure S3-2a.** Number of gastrointestinal and vomiting consultations by days since vaccination among those vaccinated and with vaccination date available (Phase III Campaign).

**Figure S3-2b.**

Number of gastrointestinal and vomiting consultations by calendar date among those living in vaccinated districts regardless of vaccination status (Phase III Campaign).

**Figure S3-3a.**

Number of hematologic and anemia consultations by days since vaccination among those vaccinated and with vaccination date available (Phase III Campaign).

**Figure S3-3b.**

Number of hematologic and anemia consultations by calendar date among those living in vaccinated districts regardless of vaccination status (Phase III Campaign).

**Figure S3-4a.**

Number of infectious and malaria consultations by days since vaccination among those vaccinated and with vaccination date available (Phase III Campaign).

**Figure S3-4b.**

Number of infectious and malaria consultations by calendar date among those living in vaccinated districts regardless of vaccination status (Phase III Campaign).

**Figure S3-5a.**

Number of neurologic and convulsions consultations by days since vaccination among those vaccinated and with vaccination date available (Phase III Campaign).

**Figure S3-5b.**

Number of neurologic and convulsions consultations by calendar date among those living in vaccinated districts regardless of vaccination status (Phase III Campaign).

**Figure S3-6a.**

Number of respiratory and cough consultations by days since vaccination among those vaccinated and with vaccination date available (Phase III Campaign).

**Figure S3-6b.**

Number of respiratory and cough consultations by calendar date among those living in vaccinated districts regardless of vaccination status (Phase III Campaign).

**Figure S3-7a.**

Number of non-specific and fever consultations by days since vaccination among those vaccinated and with vaccination date available (Phase III Campaign).

**Figure S3-7b.**

Number of non-specific and fever consultations by calendar date among those living in vaccinated districts regardless of vaccination status (Phase III Campaign).

**Figure S3-8a.**

Number of “other” and malnutrition consultations by days since vaccination among those vaccinated and with vaccination date available (Phase III Campaign).

**Figure S3-8b.**

Number of “other” and malnutrition consultations by calendar date among those living in vaccinated districts regardless of vaccination status (Phase III Campaign).
